# Supplementary material for: Fungal Diversity and Composition of the Continental Solar Saltern in Añana Salt Valley (Spain)
Source: J Fungi (Basel). 2021 Dec 14;7(12):1074. doi: 10.3390/jof7121074 (PMC8703443; doi:10.3390/jof7121074)
Supplement: Supplementary file 1 [file jof-07-01074-s001.zip › Figure S1_Table S1.pdf]

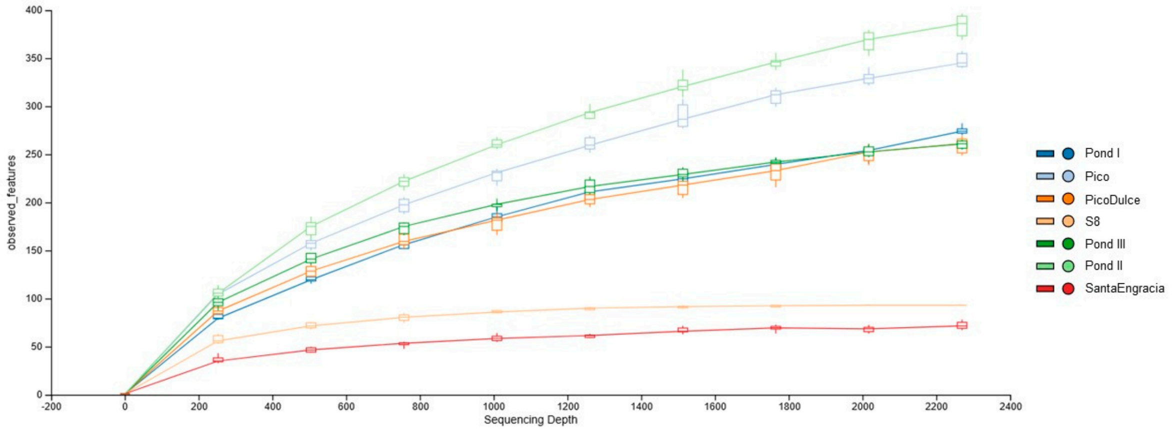


**Figure S1.** Rarefaction curves representing the ASVs (observed features) at different sequencing depths. Each curve represents an analyzed location that corresponds with the color of the legend.

**Table S1.** Sampling site information and physicochemical characteristics of the water at each location measured *in situ* at the moment of sampling.

| Sample site | Water type | Temperature (ºC) | pH | NaCl (g/L) | Conductivity (µs/cm) |
|---|---|---|---|---|---|
| SE Spring | Spring | 16.5 | 6.6 | 205 | Saturation |
| Pico Spring | Spring | 14.1 | 7.5 | 220 | Saturation |
| Pond I | Pond | 14.3 | 7.5 | 240-250 | Saturation |
| Pond II | Pond | 14.9 | 7.6 | 200 | Saturation |
| Pond III | Pond | 17.6 | 7.8 | 240-250 | Saturation |
| Pico Dulce Spring | Spring | 12.7 | 7.3 | 10.1 | 16.890 |
| S8 | Groundwater | 13.5 | 7.2 | 3.8 | 6.385 |

SE, Santa Engracia.
