# Supplementary material for: Fungal Diversity and Composition of the Continental Solar Saltern in Añana Salt Valley (Spain)
Source: J Fungi (Basel). 2021 Dec 14;7(12):1074. doi: 10.3390/jof7121074 (PMC8703443; doi:10.3390/jof7121074)
Supplement: Supplementary file 1 [file jof-07-01074-s001.zip › Table S3.pdf]

Table S3. Relative abundance (%) of the genera detected at each sampling site in the saltern of Añana Salt Valley. The results are presented classified by phylum (*Ascomycota* or *Basidiomycota*). The percentage of the unclassified ASVs at genus level at each location is also shown.

Genus	Sampling sites						
	SE	Pico	Pond I	Pond II	Pond III	Pico Dulce	S8
Ascomycota							
Absconditella	0.00	0.00	0.00	0.01	0.00	0.00	0.00
Acarospora	0.00	0.00	0.00	0.01	0.00	0.00	0.00
Acremonium	0.00	0.01	0.00	0.00	0.00	0.00	0.00
Acrospermum	0.00	0.00	0.00	0.00	0.09	0.16	0.22
Aequabiliella	0.00	0.02	0.00	0.00	0.00	0.00	0.00
Alfaria	0.00	0.00	0.00	0.00	0.73	0.00	0.00
Alternaria	0.05	0.15	0.15	2.17	2.44	0.69	0.31
Angustimassarina	0.00	0.02	0.00	0.00	0.00	0.00	0.00
Arthrinium	0.00	0.00	0.00	0.01	0.00	0.00	0.00
Arthrocatena	0.00	0.03	0.00	0.00	0.00	0.00	0.00
Articulospora	0.00	0.00	0.03	0.00	0.10	0.00	0.00
Ascochyta	0.00	0.00	0.00	0.02	0.00	0.00	0.00
Aspergillus	0.00	0.04	0.05	0.11	0.15	0.22	0.22
Bacidina	0.00	0.00	0.00	0.02	0.00	0.00	0.00
Bipolaris	0.00	0.00	0.00	0.07	0.00	0.00	0.00
Biscogniauxia	0.00	0.03	0.00	0.00	0.00	0.00	0.00
Blastobotrys	0.00	0.00	0.00	0.02	0.00	0.00	0.00
Blumeria	0.00	0.00	0.00	0.10	0.02	0.05	0.00
Boeremia	0.00	0.02	0.03	0.13	0.00	0.00	0.00
Botryotrichum	0.00	0.00	0.00	0.00	0.04	0.00	0.00
Brettanomyces	0.00	0.03	0.00	0.00	0.00	0.00	0.00
Cadophora	0.00	0.01	0.00	0.07	0.05	0.00	0.00
Candida	0.05	0.25	0.06	0.10	0.00	2.16	2.07
Catenulostroma	0.00	0.00	0.00	0.00	0.00	0.08	0.00
Chaetomium	0.00	0.00	0.00	0.00	0.01	0.00	0.00
Chalara	0.00	0.00	0.00	0.40	0.00	0.00	0.00
Chalastospora	0.00	0.00	0.00	0.01	0.00	0.00	0.00
Ciboria	0.00	0.00	0.00	0.09	0.37	0.00	0.00
Ciliophora	0.00	0.00	0.00	0.00	0.00	0.02	0.00
Citeromyces	0.00	0.00	0.00	0.00	0.00	0.05	0.00
Cladoriella	0.00	0.00	0.00	0.02	0.00	0.00	0.00
Cladosporium	0.01	0.77	1.08	2.40	2.52	2.56	2.73
Claviceps	0.00	0.00	0.00	0.05	0.00	0.00	0.00
Coelodictyosporium	0.00	0.08	0.00	0.00	0.00	0.00	0.00
Colletotrichum	0.00	0.00	0.00	0.08	0.00	0.00	0.00
Comoclathris	0.00	0.62	0.00	0.00	0.00	0.23	0.00
Coniochaeta	0.02	0.03	0.00	0.00	0.04	0.00	0.00
Conioscypha	0.00	0.00	0.00	0.01	0.00	0.00	0.00
Constantinomyces	0.00	0.00	0.01	0.00	0.11	0.00	0.00
Cordyceps	0.00	0.00	0.05	0.00	0.00	0.00	0.00
Crocicreas	0.00	0.04	0.02	0.00	0.55	0.22	0.00

Cryptocline	0.00	0.00	0.02	0.11	0.00	0.00	0.00
Cyberlindnera	0.01	0.00	0.00	0.00	0.00	0.00	0.00
Cyclaneusma	0.00	0.06	0.01	0.08	0.00	0.00	0.00
Cytospora	0.00	0.05	0.03	0.00	0.00	0.00	0.00
Debaryomyces	0.00	0.09	0.15	0.93	2.09	2.04	0.53
Dekkera	0.00	0.00	0.00	0.00	0.03	0.33	1.28
Dematiopleospora	0.00	0.02	0.00	0.00	0.00	0.03	0.00
Devriesia	0.00	0.00	0.24	0.00	0.00	0.00	0.00
Diaporthe	0.00	0.00	0.02	0.00	0.00	0.02	0.00
Didymella	0.00	0.00	0.00	0.10	0.00	1.17	1.63
Dissoconium	0.00	0.00	0.00	0.00	0.00	0.05	0.00
Dothiora	0.00	0.01	0.00	0.00	0.00	0.00	0.00
Endoconidioma	0.00	0.02	0.00	0.11	0.00	0.00	0.00
Epicoccum	0.00	0.83	0.17	7.52	7.96	0.30	0.00
Erysiphe	0.00	0.00	0.00	0.84	0.00	0.00	0.00
Evernia	0.00	0.00	0.02	0.03	0.00	0.00	0.00
Exophiala	0.00	0.00	0.06	0.14	0.00	0.00	0.00
Exserohilum	0.00	0.00	0.00	0.04	0.00	0.00	0.00
Fusariella	0.00	0.00	0.00	0.00	0.91	0.00	0.00
Fusarium	0.00	0.03	0.00	0.00	0.72	0.00	0.00
Fuscidea	0.00	0.00	0.00	0.06	0.00	0.00	0.00
Gibberella	0.00	0.00	0.00	0.00	0.00	0.00	0.00
Gibellulopsis	0.00	0.00	0.00	0.00	0.01	0.00	0.00
Golovinomyces	0.00	0.00	0.48	0.42	0.00	0.04	0.00
Gyrographa	0.00	0.00	0.00	0.03	0.00	0.00	0.00
Gyrothrix	0.00	0.00	0.00	0.05	0.00	0.00	0.00
Hanseniaspora	21.91	0.55	1.20	0.40	2.22	0.26	0.18
Hansfordia	0.00	0.06	0.07	0.25	0.07	0.00	0.00
Hormodochis	0.00	0.00	0.00	0.00	0.00	0.44	0.00
Hormonema	0.00	0.01	0.09	0.04	0.00	0.00	0.00
Hortaea	0.00	0.00	0.00	0.00	0.13	0.00	0.13
Hymenoscyphus	0.00	0.02	0.00	0.01	0.00	0.00	0.00
Hyphopichia	0.00	0.01	0.00	0.00	0.00	0.00	0.00
Hypoxylon	0.00	0.00	0.00	0.06	0.00	0.00	0.00
Hysterobrevium	0.00	0.00	0.00	0.02	0.00	0.00	0.00
Iodophanus	0.00	0.00	0.04	0.00	0.00	0.00	0.00
Issatchenkia	0.01	0.03	0.00	0.00	0.00	0.03	0.00
Kalmusia	0.00	0.00	0.00	0.06	0.00	0.00	0.00
Keissleriella	0.00	2.62	0.06	0.00	0.00	1.26	0.88
Knufia	0.00	0.05	0.46	0.09	1.76	0.00	0.00
Komagataella	0.00	0.00	0.00	0.02	0.00	0.00	0.00
Kregervanrija	0.00	0.01	0.00	0.00	0.00	0.09	0.18
Lachancea	1.69	0.00	0.17	0.00	0.00	0.00	0.00
Lachnellula	0.00	0.01	0.00	0.00	0.00	0.00	0.00
Lapidomyces	0.00	0.00	0.15	0.02	0.00	0.00	0.00
Lasiobolidium	0.00	0.01	0.00	0.00	0.00	0.00	0.00
Lecania	0.00	0.00	0.02	0.00	0.03	0.00	0.00
Lecanicillium	0.00	0.00	0.00	0.01	0.00	0.01	0.00

Lecanora	0.00	0.00	0.06	0.59	0.00	0.00	0.00
Lepraria	0.00	0.00	0.00	0.06	0.00	0.00	0.00
Leptobacillium	0.00	0.00	0.00	0.00	0.00	0.04	0.00
Leptosillia	0.00	0.01	0.00	0.00	0.00	0.00	0.00
Leptospora	0.00	0.00	0.22	0.00	0.00	0.00	0.00
Leveillula	0.00	0.00	0.04	0.09	0.00	0.00	0.00
Libertasomyces	0.00	0.02	0.00	0.00	0.00	0.02	0.00
Lophodermium	0.00	0.00	0.00	0.25	0.00	0.00	0.00
Lophotrichus	0.00	0.00	0.00	0.02	0.00	0.00	0.00
Metschnikowia	5.94	0.14	0.25	0.23	0.55	0.00	0.00
Meyerozyma	0.00	0.00	0.00	0.00	0.16	0.01	0.00
Microascospora	0.00	0.03	0.01	0.00	0.00	0.00	0.00
Mollisia	0.00	0.05	0.00	0.00	0.00	0.00	0.00
Monocillium	0.00	0.01	0.00	0.00	0.00	0.00	0.00
Monographella	0.00	0.00	0.03	0.04	0.08	0.04	0.00
Mycoarthris	0.00	0.00	0.00	0.02	0.00	0.00	0.00
Mycosphaerella	0.41	0.82	1.74	3.54	3.76	1.53	3.25
Myrmecridium	0.00	0.00	0.00	0.01	0.00	0.00	0.00
Nakazawaea	0.00	0.00	0.00	0.01	0.00	0.00	0.00
Neoascochyta	0.00	0.15	0.44	0.15	0.00	0.24	0.35
Neobulgaria	0.00	0.00	0.05	0.00	0.00	0.00	0.00
Neocamarosporium	0.00	0.00	0.04	0.22	0.87	0.00	0.00
Neocatenulostroma	0.00	0.00	0.00	0.07	0.09	0.00	0.00
Neocucurbitaria	0.00	0.02	0.02	0.00	0.00	0.02	0.00
Neodevriesia	0.00	0.05	0.09	0.28	0.00	0.20	0.26
Neoerysiphe	0.00	0.00	0.00	0.00	0.00	0.06	0.13
Neophaeomoniella	0.00	0.14	0.00	0.01	0.14	0.00	0.00
Neophysalospora	0.00	0.38	0.27	0.47	0.24	0.00	0.00
Neosetophoma	0.00	0.02	0.05	0.00	0.17	0.03	0.00
Niesslia	0.00	0.00	0.15	0.00	0.00	0.00	0.00
Nigrosabulum	0.00	0.00	0.00	0.04	0.00	0.00	0.00
Nigrospora	0.00	0.00	0.00	0.08	0.00	0.00	0.00
Paecilomyces	0.00	0.00	0.00	0.00	0.00	0.00	0.00
Paramycosphaerella	0.01	0.00	0.00	0.00	0.00	0.00	0.00
Paraphaeosphaeria	0.00	0.00	0.00	0.02	0.00	0.00	0.00
Paraphoma	0.00	0.00	0.00	0.00	0.00	0.00	0.00
Parastagonospora	0.00	0.00	0.03	0.01	0.00	0.02	0.00
Penicillium	0.00	0.07	1.48	0.08	0.39	0.05	0.35
Perusta	0.00	0.00	0.00	0.12	0.17	0.00	0.00
Petrophila	0.00	0.00	0.00	0.00	0.04	0.00	0.00
Pezicula	0.00	0.02	0.00	0.00	0.00	0.00	0.00
Phaeococcomyces	0.00	0.01	0.03	0.24	0.93	0.01	0.00
Phaeophleospora	0.00	0.00	0.00	0.00	0.00	0.01	0.00
Phaeosphaeria	0.00	0.06	0.29	1.04	0.72	0.00	0.00
Phaeotheca	0.00	0.17	0.00	0.08	0.67	0.00	0.00
Phialocephala	0.00	0.00	0.00	0.03	0.00	0.00	0.00
Phomatodes	0.00	0.00	0.06	0.00	0.00	0.00	0.00
Phomatospora	0.00	0.00	0.00	0.00	0.15	0.00	0.00

Physcia	0.00	0.17	0.43	1.23	0.62	0.00	0.00
Pichia	0.05	0.00	0.00	0.00	0.00	2.71	12.62
Pilidium	0.00	0.00	0.00	0.03	0.00	0.00	0.00
Platystomum	0.00	0.01	0.00	0.00	0.00	0.00	0.00
Plectosphaerella	0.00	0.00	0.60	0.15	0.00	0.10	0.18
Podosphaera	0.00	0.00	0.08	0.31	0.00	0.00	0.00
Polycauliona	0.00	0.00	0.22	0.00	0.00	0.00	0.00
Polyscytalum	0.00	0.00	0.02	0.00	0.00	0.00	0.00
Pringsheimia	0.00	0.16	0.00	0.00	0.00	0.13	0.00
Proliferodiscus	0.00	0.03	0.00	0.00	0.00	0.00	0.00
Propolis	0.00	0.00	0.00	0.02	0.01	0.00	0.00
Protomyces	0.00	0.02	0.02	0.00	0.00	0.14	0.00
Pseudocoleophoma	0.00	0.00	0.00	0.01	0.00	0.00	0.00
Pseudoneoconiothyrium	0.00	0.00	0.00	0.05	0.00	0.00	0.00
Pseudoophiobolus	0.00	0.00	0.01	0.00	0.00	0.03	0.00
Pyrenopeziza	0.00	0.03	0.04	0.00	0.00	0.00	0.00
Pyrenophora	0.00	0.00	0.00	0.01	0.00	0.18	0.00
Rachicladosporium	0.00	0.00	0.00	0.00	0.00	0.01	0.00
Radulidium	0.00	0.00	0.00	0.00	0.00	0.00	0.00
Ramichloridium	0.00	0.00	0.00	0.00	0.00	0.02	0.00
Ramularia	0.00	0.04	0.19	0.30	0.00	2.49	7.56
Rhytisma	0.00	0.03	0.00	0.12	0.00	0.00	0.00
Rinodina	0.00	0.06	0.00	0.00	0.00	0.00	0.00
Rosasphaeria	0.00	0.00	0.03	0.00	0.00	0.00	0.00
Saccharomyces	49.89	8.38	5.84	1.49	20.13	2.03	3.25
Saccharomycodes	0.50	0.00	0.00	0.00	0.00	0.00	0.00
Saccharomycopsis	0.00	0.00	0.00	0.02	0.00	0.00	0.00
Saccothecium	0.00	0.00	0.00	0.00	0.00	0.03	0.00
Sarocladium	0.00	0.42	0.42	0.54	0.81	0.00	0.00
Saxophila	0.02	0.00	0.04	0.00	1.64	0.00	0.00
Schwanniomyces	0.02	0.03	0.00	0.02	0.06	0.00	0.00
Scoliciosporum	0.00	0.00	0.00	0.14	0.00	0.00	0.00
Selenophoma	0.00	0.02	0.00	0.00	0.00	0.00	0.00
Septoriella	0.00	0.00	0.00	0.21	0.00	0.00	0.00
Simplicillium	0.00	0.00	0.00	0.00	0.00	0.08	0.00
Sparticola	0.00	0.00	0.03	0.00	0.00	0.00	0.00
Sphaerulina	0.01	0.00	0.00	0.07	0.00	0.00	0.00
Spissiomyces	0.00	0.02	0.00	0.02	0.00	0.00	0.00
Stachybotrys	0.00	0.00	0.00	0.00	0.00	0.01	0.00
Starmerella	0.00	0.00	0.00	0.00	0.00	0.02	0.00
Stemphylium	0.02	0.00	0.34	0.70	0.38	3.52	3.69
Subplenodomus	0.00	0.00	0.11	0.00	0.00	0.00	0.00
Symbiotaphrina	0.00	0.04	0.01	0.00	0.00	0.00	0.00
Talaromyces	0.00	0.00	0.23	0.01	0.00	0.00	0.00
Tamaricicola	0.00	0.00	0.00	0.01	0.06	0.00	0.00
Taphrina	0.00	0.22	0.32	3.63	0.86	0.04	0.00
Teratosphaeria	0.00	0.00	0.12	0.01	0.00	0.00	0.00
Tetracladium	0.00	0.00	0.00	0.00	0.00	0.01	0.00

Thyronectria	0.00	0.00	0.05	0.00	0.00	0.00	0.00
Tiarosporella	0.00	0.00	0.00	0.00	0.00	0.02	0.00
Tilachlidium	0.00	0.00	0.00	0.02	0.00	0.00	0.00
Torulaspora	0.22	0.00	0.00	0.00	0.00	0.00	0.00
Tremateia	0.00	0.00	0.00	0.00	0.00	0.04	0.00
Trichoderma	0.00	0.00	0.00	0.01	0.00	0.00	0.00
Trichopezizella	0.00	0.03	0.00	0.00	0.00	0.00	0.00
Trichothecium	0.00	0.00	0.00	0.01	0.00	0.00	0.00
Trigonopsis	0.04	0.00	0.00	0.00	0.00	0.00	0.00
Trimmatostroma	0.00	0.72	0.41	0.22	4.97	0.02	0.00
Uzbekistanica	0.00	0.06	0.00	0.00	0.00	0.00	0.00
Valsa	0.00	0.03	0.02	0.00	0.00	0.00	0.00
Vermiconia	0.00	0.00	0.03	0.00	0.01	0.00	0.00
Veronaea	0.00	0.00	0.02	0.00	0.00	0.00	0.00
Verrucaria	0.00	0.00	0.00	0.01	0.00	0.01	0.00
Verticillium	0.00	0.00	0.00	0.00	0.05	0.00	0.00
Violella	0.00	0.00	0.00	0.04	0.00	0.00	0.00
Wickerhamiella	0.00	0.00	0.00	0.00	0.00	0.02	0.00
Wickerhamomyces	0.06	0.00	0.00	0.26	0.13	0.00	0.00
Xenocylindrosporium	0.00	0.14	0.00	0.05	0.00	0.00	0.00
Xenoteratosphaeria	0.00	0.00	0.00	0.07	0.00	0.00	0.00
Yamadazyma	0.01	0.00	0.03	0.04	0.00	0.00	0.00
Yarrowia	0.00	0.00	0.00	0.00	0.00	0.07	0.00
Zygosaccharomyces	0.09	0.00	0.00	0.00	0.00	0.00	0.00
Zygotorulaspora	0.01	0.00	0.00	0.00	0.00	0.00	0.00
Zymoseptoria	0.00	0.23	0.00	0.02	0.00	0.00	0.00
Basidiomycota							
Cinereomyces	0.00	0.01	0.00	0.00	0.00	0.00	0.09
Amphinema	0.00	0.00	0.00	0.00	0.00	0.00	0.00
Ampulloclitocybe	0.00	0.06	0.00	0.00	0.00	0.00	0.00
Amylocorticium	0.00	0.09	0.00	0.00	0.00	0.00	0.00
Amyloporia	0.00	0.08	0.00	0.03	0.00	0.00	0.00
Amylostereum	0.00	0.08	0.00	0.03	0.00	0.00	0.00
Amyloxenasma	0.00	0.01	0.01	0.00	0.04	0.00	0.00
Anomoloma	0.00	0.02	0.00	0.00	0.00	0.00	0.00
Antrodia	0.00	0.03	0.12	0.00	0.04	0.00	0.00
Antrodiella	0.00	0.34	0.00	0.05	0.00	0.00	0.00
Armillaria	0.00	0.00	0.00	0.13	0.00	0.00	0.00
Artomyces	0.00	0.02	0.00	0.00	0.00	0.00	0.00
Athelia	0.00	0.02	0.10	0.07	0.21	0.00	0.00
Auricularia	0.00	0.00	0.00	0.05	0.00	0.00	0.00
Auriporia	0.00	0.04	0.00	0.00	0.00	0.00	0.00
Baeospora	0.00	1.60	0.26	0.05	0.06	0.00	0.00
Bannozyma	0.00	0.00	0.00	0.40	0.00	0.00	0.00
Bensingtonia	0.00	0.03	0.03	0.00	0.00	0.00	0.00
Bjerkandera	0.00	0.21	0.08	0.10	0.16	0.00	0.00
Brunneoporus	0.00	0.00	0.00	0.02	0.00	0.00	0.00

Buckleyzyma	0.00	0.00	0.00	0.02	0.00	0.02	0.00
Bullera	0.00	0.02	0.00	0.00	0.00	0.00	0.00
Byssomerulius	0.00	0.13	0.00	0.00	0.03	0.00	0.00
Cabalodontia	0.00	0.05	0.00	0.00	0.09	0.00	0.00
Campanella	0.00	0.03	0.00	0.00	0.00	0.00	0.00
Carcinomyces	0.00	0.00	0.00	0.03	0.00	0.00	0.00
Cartilosoma	0.00	0.02	0.00	0.00	0.00	0.00	0.00
Ceriporia	0.00	0.08	0.01	0.00	0.15	0.00	0.00
Ceriporiopsis	0.00	0.21	0.06	0.01	0.00	0.00	0.00
Chondrostereum	0.00	0.04	0.00	0.00	0.00	0.00	0.00
Clitocybe	0.00	0.00	0.00	0.46	0.08	0.00	0.00
Coniophora	0.00	0.00	0.00	0.05	0.00	0.00	0.00
Coprinellus	0.03	0.14	0.03	0.00	0.13	0.00	0.00
Coprinopsis	0.00	0.00	0.00	0.00	0.04	0.00	0.00
Coronicium	0.00	0.01	0.00	0.00	0.00	0.00	0.00
Corticium	0.00	0.19	0.05	0.00	0.00	0.00	0.00
Crepidotus	0.00	0.02	0.01	0.00	0.00	0.00	0.00
Crinipellis	0.00	0.05	0.03	0.00	0.00	0.00	0.00
Cryptococcus	0.00	0.00	0.00	0.02	0.13	0.00	0.00
Cylindrobasidium	0.00	0.04	0.00	0.00	0.00	0.00	0.00
Cystobasidium	0.00	0.00	0.00	0.10	0.09	0.05	0.00
Cystofilobasidium	0.00	0.00	0.25	0.20	0.00	0.00	0.00
Deconica	0.00	0.00	0.00	0.00	0.06	0.00	0.00
Dichomitus	0.00	0.05	0.00	0.00	0.00	0.00	0.00
Dioszegia	0.01	0.29	0.27	0.00	0.07	0.00	0.00
Diplomitoporus	0.00	0.00	0.04	0.00	0.00	0.00	0.00
Erythrobasidium	0.00	0.00	0.00	0.03	0.00	0.00	0.00
Exidia	0.00	0.05	0.03	0.00	0.00	0.00	0.00
Exobasidium	0.00	0.00	0.00	0.03	0.00	0.00	0.00
Fellomyces	0.00	0.00	0.00	0.05	0.00	0.00	0.00
Fibroporia	0.00	0.03	0.00	0.02	0.00	0.00	0.00
Filobasidium	0.01	0.18	18.14	9.98	0.96	0.43	0.00
Flammulina	0.00	0.00	0.02	0.00	0.00	0.00	0.00
Fomes	0.00	0.05	0.04	0.00	0.00	0.00	0.00
Fomitopsis	0.00	0.06	0.00	0.00	0.00	0.00	0.00
Fonsecazyma	0.00	0.00	0.00	0.11	0.00	0.00	0.00
Fuscoporia	0.00	0.59	0.08	0.01	0.00	0.00	0.00
Galzinia	0.00	0.05	0.00	0.00	0.00	0.00	0.00
Genolevuria	0.00	0.02	0.00	0.00	0.00	0.00	0.00
Gloeocystidiellum	0.00	0.02	0.00	0.00	0.00	0.00	0.00
Gloeophyllum	0.00	0.03	0.00	0.06	0.00	0.00	0.00
Gloeoporus	0.00	0.02	0.00	0.01	0.00	0.00	0.00
Gloiothele	0.00	0.02	0.00	0.07	0.00	0.00	0.00
Gymnopus	0.00	0.05	0.00	0.01	0.05	0.00	0.00
Hebeloma	0.00	0.00	0.00	0.00	0.05	0.00	0.00
Heterobasidion	0.00	0.00	0.00	0.17	0.08	0.00	0.00
Hohenbuehelia	0.00	0.00	0.01	0.00	0.00	0.00	0.00
Holtermanniella	0.00	0.00	0.47	0.04	0.10	0.00	0.00

Hymenochaete	0.00	0.05	0.00	0.00	0.00	0.00	0.00
Hymenochaetopsis	0.00	0.14	0.12	0.00	0.08	0.00	0.00
Hyphoderma	0.00	0.07	0.03	0.00	0.07	0.00	0.00
Hyphodermella	0.00	0.22	0.05	0.05	0.00	0.00	0.00
Hyphodontia	0.00	0.28	0.00	0.02	0.00	0.00	0.00
Hypholoma	0.00	0.04	0.00	0.08	0.00	0.00	0.00
Inonotus	0.00	0.00	0.00	0.00	0.01	0.00	0.00
Irpex	0.00	0.08	0.10	0.00	0.17	0.00	0.00
Itersonilia	0.00	0.00	0.23	0.00	0.11	0.10	0.00
Kondoa	0.00	0.09	0.08	0.16	0.33	0.05	0.00
Lentinus	0.00	0.10	0.00	0.00	0.00	0.00	0.00
Lenzites	0.00	0.10	0.00	0.00	0.00	0.00	0.00
Lepista	0.00	0.00	0.00	0.02	0.00	0.00	0.00
Leptoporus	0.00	0.00	0.00	0.05	0.09	0.00	0.00
Leucogyrophana	0.00	0.01	0.00	0.00	0.00	0.00	0.00
Leucopaxillus	0.00	0.04	0.00	0.00	0.00	0.00	0.00
Leucosporidium	0.00	0.00	0.00	0.02	0.00	1.43	1.06
Limonomyces	0.00	0.01	0.01	0.00	0.00	0.00	0.00
Lyomyces	0.00	0.00	0.00	0.00	0.00	0.00	0.00
Malassezia	0.03	0.00	0.03	0.04	0.04	0.02	0.00
Marasmiellus	0.00	0.01	0.00	0.00	0.00	0.00	0.00
Marasmius	0.00	0.00	0.00	0.00	0.05	0.00	0.00
Mortierella	0.00	0.00	0.00	0.00	0.00	0.06	0.00
Mycena	0.00	0.02	0.00	0.00	0.01	0.02	0.00
Mycetinis	0.00	0.00	0.01	0.00	0.00	0.00	0.00
Mycoacia	0.00	0.04	0.01	0.00	0.00	0.00	0.00
Myxarium	0.00	0.01	0.00	0.00	0.00	0.00	0.00
Naganishia	0.00	0.08	0.04	0.00	0.05	0.05	0.00
Odonticium	0.00	0.00	0.01	0.00	0.00	0.00	0.00
Papiliotrema	0.00	0.00	0.02	0.00	0.00	0.00	0.00
Peniophora	0.00	4.25	0.91	0.37	1.49	0.04	0.00
Peniophorella	0.00	0.17	0.00	0.11	0.07	0.02	0.00
Phaeolus	0.00	0.00	0.00	0.03	0.00	0.00	0.00
Phaeomarasmius	0.00	0.04	0.04	0.00	0.00	0.00	0.00
Phanerochaete	0.00	0.16	0.04	0.07	0.00	0.00	0.00
Phellinus	0.00	0.00	0.00	0.00	0.04	0.00	0.00
Phlebia	0.00	2.33	0.39	0.53	0.19	0.00	0.00
Phlebiopsis	0.00	0.13	0.03	0.03	0.23	0.00	0.00
Pholiota	0.01	0.00	0.00	0.00	0.00	0.00	0.00
Phylloporia	0.00	0.02	0.00	0.00	0.00	0.00	0.00
Pilatoporus	0.00	0.04	0.00	0.00	0.00	0.00	0.00
Pleurotus	0.00	0.04	0.07	0.04	0.59	0.00	0.00
Porostereum	0.00	0.02	0.00	0.04	0.00	0.00	0.00
Postia	0.00	0.02	0.00	0.00	0.00	0.00	0.00
Psathyrella	0.00	0.14	0.03	0.04	0.40	0.00	0.00
Pseudoclitocybe	0.00	0.00	0.00	0.00	0.04	0.00	0.00
Radulomyces	0.00	0.04	0.00	0.00	0.00	0.00	0.00
Resinicium	0.00	1.18	0.30	0.04	0.45	0.00	0.00

Resupinatus	0.00	1.16	0.10	0.11	0.07	0.00	0.00
Rhizophlyctis	0.00	0.00	0.00	0.00	0.09	0.00	0.00
Rhodocollybia	0.00	0.00	0.00	0.01	0.00	0.00	0.00
Rhodosporidiobolus	0.00	0.00	0.00	0.11	0.02	0.00	0.00
Rhodotorula	0.00	0.17	0.00	0.13	0.00	0.00	0.00
Rugosomyces	0.00	0.03	0.00	0.00	0.00	0.00	0.00
Schizophyllum	0.00	0.67	0.39	0.01	0.16	0.00	0.00
Scopuloides	0.00	0.10	0.03	0.01	0.00	0.00	0.00
Septobasidium	0.00	0.00	0.00	0.03	0.00	0.00	0.00
Sidera	0.00	0.82	0.15	0.10	0.24	0.00	0.00
Singerocybe	0.00	0.01	0.00	0.00	0.00	0.00	0.00
Sistotrema	0.00	0.02	0.01	0.00	0.00	0.00	0.00
Sistotremastrum	0.00	0.83	0.21	0.10	0.00	0.00	0.00
Skeletocutis	0.00	0.12	0.03	0.01	0.00	0.00	0.00
Sporobolomyces	0.00	0.34	0.06	0.13	0.11	0.52	1.80
Steccherinum	0.00	0.44	0.03	0.05	0.00	0.00	0.00
Stereum	0.00	2.83	1.95	0.82	1.66	0.00	0.00
Stropharia	0.00	0.00	0.03	0.00	0.00	0.00	0.00
Suillus	0.00	0.02	0.00	0.02	0.00	0.00	0.00
Symmetrospora	0.00	0.08	0.00	0.12	0.00	0.09	0.00
Tausonia	0.00	0.00	0.00	0.00	0.00	0.08	1.54
Terana	0.00	0.02	0.00	0.00	0.00	0.00	0.00
Thanatephorus	0.00	0.00	0.00	0.00	0.00	0.02	0.00
Trametes	0.00	1.77	0.48	0.22	1.07	0.45	0.53
Trechispora	0.00	0.14	0.01	0.01	0.06	0.00	0.00
Tremella	0.00	0.00	0.00	0.07	0.00	0.00	0.00
Trichaptum	0.00	0.02	0.02	0.04	0.02	0.00	0.00
Tricholoma	0.00	0.02	0.00	0.00	0.00	0.00	0.00
Tricholomopsis	0.00	0.06	0.00	0.04	0.00	0.00	0.00
Trichosporon	0.00	0.00	0.04	0.00	0.00	0.00	0.00
Tubaria	0.00	0.00	0.07	0.00	0.00	0.00	0.00
Tubulicrinis	0.00	0.27	0.02	0.03	0.00	0.00	0.00
Typhrasa	0.00	0.00	0.00	0.02	0.06	0.00	0.00
Typhula	0.00	0.29	0.00	0.05	0.00	0.00	0.00
Udeniomyces	0.00	0.00	0.08	0.06	0.66	0.02	0.00
Uncobasidium	0.00	0.00	0.05	0.00	0.00	0.00	0.00
unidentified	0.00	0.00	0.00	0.00	0.00	0.00	0.00
unidentified	0.00	0.00	0.02	0.00	0.00	0.00	0.00
Vishniacozyma	0.06	0.71	11.24	4.27	0.75	18.37	24.19
Vuilleminia	0.00	0.40	0.19	0.05	0.11	0.00	0.00
Wallemia	0.02	0.10	24.04	0.01	0.18	0.00	0.00
Wolfiporia	0.00	0.03	0.00	0.00	0.00	0.00	0.00
Xylobolus	0.00	0.01	0.00	0.00	0.00	0.00	0.00
Xylodon	0.00	0.08	0.01	0.00	0.17	0.00	0.00
**Unclassified**	18.79	53.58	17.99	43.76	25.44	51.58	28.63

SE, Santa Engracia.
