# Supplementary material for: Fungal Diversity and Composition of the Continental Solar Saltern in Añana Salt Valley (Spain)
Source: J Fungi (Basel). 2021 Dec 14;7(12):1074. doi: 10.3390/jof7121074 (PMC8703443; doi:10.3390/jof7121074)
Supplement: Supplementary file 1 [file jof-07-01074-s001.zip › Table S4_Species.pdf]

Table S4. Species identified at the locations analyzed in the saltern. Species with a relative abundance >0.5% are shown.

Sample site	Species	Relative abundance (%)
SE Spring	*Saccharomyces bayanus*	3.4
	Metschnikowia shanxiensis	2.7
	Lachancea quebecensis	1.7
	Metschnikowia rubicola	0.6
	Saccharomycodes ludwigii	0.5
Pico Spring	*Stereum hirsutum*	1.2
	Resupinatus europaeus	1.1
	Keissleriella rosarum	1
	Epicoccum dendrobii	0.8
	Mycosphaerella tassiana	0.8
	Resinicium bicolor	0.8
	Stereum subtomentosum	0.7
	Trimmatostroma salinum	0.7
	Schizophyllum commune	0.7
	Cladosporium delicatulum	0.6
	Sidera vulgaris	0.6
	Sistotremastrum niveocremeum	0.5
Pond I	*Wallemia ichthyophaga*	24
	Vishniacozyma victoriae	10.5
	Mycosphaerella tassiana	1.7
	Cladosporium delicatum	1
	Stereum hirsutum	0.7
Pond II	*Epicoccum dendrobii*	6.5
	Mycosphaerella tassiana	3.5
	Vishniacozyma victoriae	2.8
	Cladosporium delicatulum	2.3
	Taphrina carpini	1.7
	Physcia dubia	1.2
	Phaeosphaeria caricicola	1
	Vishniacozyma carnescens	0.8
	Lecanora compallens	0.6
	Neophysalospora eucalypti	0.5
	Clitocybe nebularis	0.5
Pond III	*Epicoccum dendrobii*	7.7
	Trimmatostroma salinum	5
	Mycosphaerella tassiana	3.8
	Cladosporium delicatulum	2.1
	Saxophila tyrrhenica	1.6
	Knufia mediterranea	1.3
	Neocamarosporium lamiacearum	0.9
	Stereum hirsutum	0.8
	Phaeosphaeria caricicola	0.7
	Udeniomyces pyricola	0.7
	Physcia dubia	0.6
	Crocicreas pallidum	0.6
	Metschnikowia shanxiensis	0.5
Pico Dulce Spring	*Pichia mandshurica*	2.7
	Cladosporium delicatulum	2.6
	Mycosphaerella tassiana	1.5
	Vishniacozyma victoriae	1.2

	Keissleriella rosarum	1.1
	Candida sake	1
	Candida zeylanoides	0.9
	Sporobolomyces roseus	0.5
	Vishniacozyma carnescens	0.5
	Trametes versicolor	0.5
S8	*Pichia mandshurica*	12
	Mycosphaerella tassiana	3.3
	Cladosporium delicatulum	2.7
	Sporobolomyces roseus	1.8
	Tausonia pullulans	1.5
	Dekkera custersiana	1.3
	Vishniacozyma victoriae	1
	Candida hyderabadensis	0.9
	Saccharomyces bayanus	0.7
	Pichia membranifaciens	0.7
	Keissleriella rosarum	0.7
	Candida tropicalis	0.6
	Candida zeylanoides	0.5
	Trametes versicolor	0.5
	Vishniacozyma carnescens	0.5

SE, Santa Engracia
