# Supplementary figures and images for: Fungal Diversity and Composition of the Continental Solar Saltern in Añana Salt Valley (Spain)
Source: J Fungi (Basel). 2021 Dec 14;7(12):1074. doi: 10.3390/jof7121074 (PMC8703443; doi:10.3390/jof7121074)

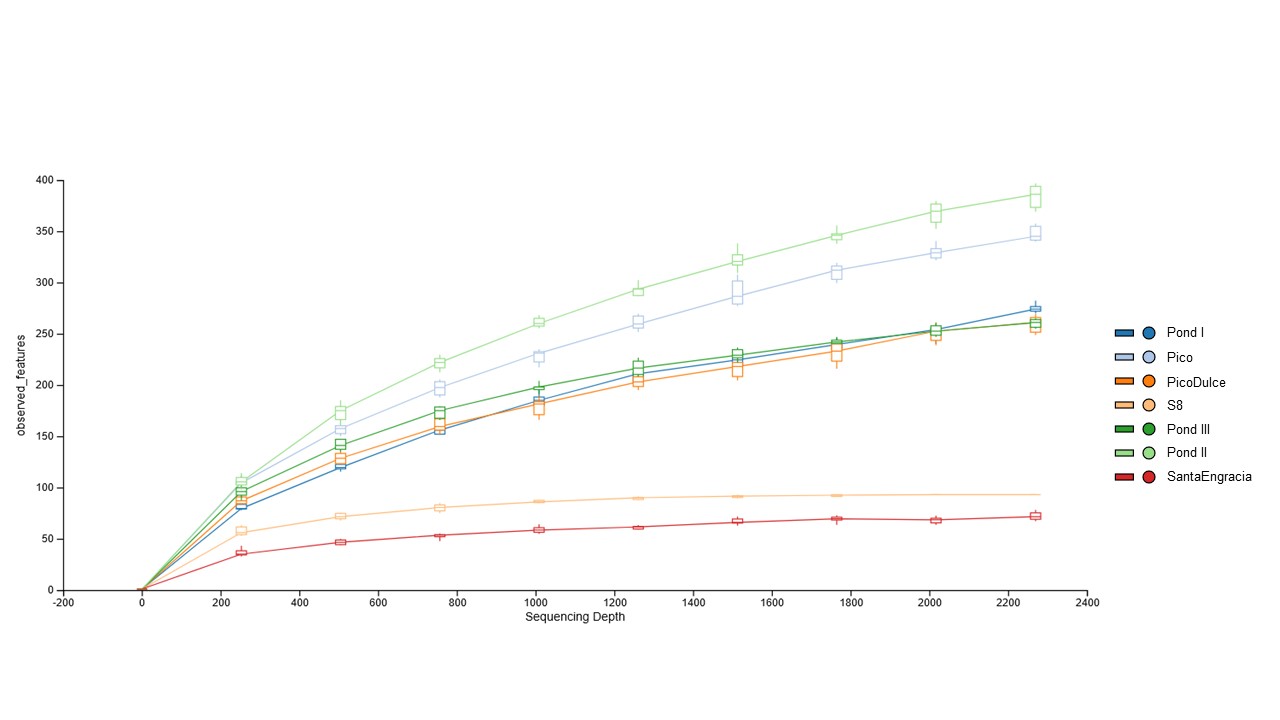

Supplement: Supplementary file 1 [file jof-07-01074-s001.zip › Figure S1_Rarefaction_curves.jpg]
